# Real-World Evidence Assessing the Safety of Administering Intravenous Rituximab Biosimilar in the First Cycle and Subcutaneous Rituximab in Subsequent Cycles in B-Cell Lymphoma Patients

**DOI:** 10.3390/pharmacy13030083

**Published:** 2025-06-11

**Authors:** Tamather Almandeel, Mansoor Ahmed Khan, Ashwag Algethami, Mashael S. Alaboud, Munirah A. Alkathiri, Mohammed Aseeri, Ahmed Absi, Mubarak Almansour, Abdullah Alotaibi

**Affiliations:** 1 Pharmaceutical Care Department, King Abdulaziz Medical City, Jeddah 21423, Saudi Arabia; tamather.khalid2@gmail.com (T.A.); aalgethami16@gmail.com (A.A.); m.alaboud93@hotmail.com (M.S.A.); munirahaalkathiri@gmail.com (M.A.A.); aseerima@mngha.med.sa (M.A.); 2 College of Medicine, King Saud bin Abdulaziz University for Health Sciences, Jeddah 21423, Saudi Arabia; absiaa@mngha.med.sa (A.A.); mansourmu@mngha.med.sa (M.A.); 3 King Abdullah International Medical Research Center, Jeddah 21423, Saudi Arabia; 4 Oncology Department, King Abdulaziz Medical City, Jeddah 21423, Saudi Arabia; 5 Pharmaceutical Care Department, Maternal and Children Specialist Hospital, Jeddah 23816, Saudi Arabia; abdullah.fa.94@gmail.com

**Keywords:** oncology biosimilars, rituximab biosimilar, extrapolation, pharmacovigilance, real-world evidence

## Abstract

**Background:** Biosimilar versions of rituximab have similar safety and efficacy as the reference product across all indications based on the extrapolation principle. Our organization replaced intravenous (IV) rituximab (Mabthera) with IV rituximab (Truxima-Biosimilar) in 2021. Hence, our practice changed to providing first cycles of IV rituximab (Truxima-Biosimilar) instead of rituximab (Mabthera), and if the first cycle was completed without severe infusion-related reactions (IRRs), then subsequent cycles were given with subcutaneous (SC) rituximab as per institutional guidelines. However, the safety of this approach has not been evaluated. **Methods:** A retrospective study was conducted at the Princess Nourah Oncology Center in Saudi Arabia. The primary objective was to assess IRRs after using IV rituximab (Truxima-Biosimilar) in the first cycle followed by SC rituximab in subsequent cycles. **Results:** Of the 71 patients reviewed, 35 patients met the eligibility criteria. Only one (3%) patient developed an IRR. However, it was a Grade 1 IRR, as per CTCAE.V5, and the patient was able to complete the remaining IV infusion successfully. Hence, all patients transitioned from IV rituximab biosimilar to SC rituximab Mabthera. **Conclusions:** This real-world study demonstrates that transitioning from IV rituximab biosimilar to SC Mabthera is a well-tolerated and safe practice, confirming the extrapolation principle of biosimilars.

## 1. Introduction

The introduction of biosimilars is a cost-effective strategy to provide an alternative for a reference product [1]. The integration of biosimilars into clinical practice in the Kingdom of Saudi Arabia (KSA) befits Saudi Vision 2030. The KSA is the largest biosimilar market in the Middle East and Africa [2]. A local simulation study evaluated cost efficiency and expanded access to care by switching from reference filgrastim and pegfilgrastim to biosimilar filgrastim in 4000 patients in the country. Biosimilar conversion from the reference to biosimilar filgrastim enabled expanded access to ado-trastuzumab emtansine, ranging from 61 to 191 patients with locally advanced HER2/neu-positive breast cancer in adjuvant settings [3].

The Saudi Food and Drug Authority (SFDA) has approved many biosimilars for monoclonal antibodies such as rituximab, trastuzumab, and bevacizumab. The rituximab biosimilar was implemented only partially in our organization, mainly in in-patient regimens for treating malignant and non-malignant conditions. Subcutaneous (SC) rituximab was continued in the formulary, as there is a major advantage to using SC rituximab regarding ease and convenience of administration. Therefore, our organization decided to limit IV rituximab biosimilar use to B-cell acute lymphocytic leukemia, salvage regimens for lymphomas, chronic lymphocytic leukemia, the first cycle for lymphoma patients, aiming for SC rituximab in subsequent cycles in an out-patient setting, and all non-malignant conditions [1,2].

Rituximab is an anti-CD20 monoclonal antibody that is highly effective in treating B-cell malignancies. It has also shown efficacy in autoimmune disorders like rheumatoid arthritis and granulomatosis with polyangiitis. Rituximab’s mechanism of action involves the depletion of B-cells through complement-dependent cytotoxicity (CDC) and antibody-dependent cell-mediated cytotoxicity (ADCC) [4].

The originator rituximab (MabThera) was first approved by the Food and Drug Administration (FDA) in 1997, followed by the European Medicines Agency (EMA) in 1998, and the Saudi Food and Drug Authority (SFDA) in 2008. Later, with the expiration of rituximab’s patent, Truxima (rituximab-abbs) became the first biosimilar, receiving FDA approval on 28 November 2018 [5]. This was followed by the approval of Ruxience (rituximab-pvvr) in 2019 and Riabni (rituximab-arrx) in 2020 [6,7].

Several studies have demonstrated that rituximab biosimilars offer equivalent efficacy and safety compared to the originator rituximab [8,9]. In the REFLECT trial, Riximyo (a rituximab biosimilar) was combined with R-CHOP in CD20-positive DLBCL. A 94.7% overall response rate (ORR) was achieved in this trial; 65% of patients achieved a complete response (CR) and 30% had a partial response (PR). The one-year progression-free survival rate was 84.9%, while 78% successful PFS was obtained after two years. The safety data indicated that adverse events were suffered by 84.6% of patients. Serious adverse events (SAEs) occurred in 37% of cases [8]. Similarly, in the ASSIST-FL study, patients with untreated advanced follicular lymphoma (FL) received rituximab biosimilar Rixathon (GP2013) and CVP (cyclophosphamide, vincristine, and prednisone) [9]. The study established that Rixathon was as equally safe and effective as the originator rituximab. In the test group, the ORR was 87.1% for Rixathon, compared to 87.5% for the originator, meeting its primary endpoint of equivalence. In addition, the safety data pointed to a similar profile across both groups [9].

Rituximab is a key therapy used in the treatment of B-cell lymphomas and can be administered either intravenously (IV) or subcutaneously (SC). Although both formulations have shown similar efficacy, there are important differences in their pharmacokinetics, safety, route of administration, and resource use [9]. Clinical trials have shown that SC rituximab is non-inferior to IV rituximab in terms of efficacy in a follicular lymphoma SABRINA trial and in a diffuse large B-cell lymphoma (DLBCL) MabEase trial [10,11]. Pharmacokinetically, SC rituximab achieves higher and more sustained serum trough levels due to its slower absorption, whereas IV administration results in a rapid peak concentration, but similar overall drug exposure [12,13,14]. The SABRINA trial showed that SC rituximab was non-inferior to IV rituximab in follicular lymphoma patients, with an overall response rate (ORR) of 84.9% with SC administration and 84.4% with IV use. Progression-free survival (PFS) was similar in both groups [10]. In contrast, the MabEase study showed that in a diffuse large B-cell lymphoma (DLBCL) setting, SC rituximab was as equally efficacious as IV rituximab, with ORRs of 90.5% and 84.4%, respectively. Response rates for complete responses were also similar [11].

Safety profiles between the two formulations are comparable, although IV rituximab is associated with a higher incidence of infusion-related reactions (IRRs), particularly during the first infusion, necessitating premedication, extended infusion time, and monitoring. In contrast, SC administration offers a significant advantage in terms of patient convenience, as it is administered over 5–7 min, thereby reducing bed time and healthcare resource utilization [15,16].

Biosimilars have the potential to offer cost savings with comparable efficacy and safety to innovator products, and thus, to increase access to treatment for more patients. One of our recent papers showed the significant cost impact after the formulary substitution of rituximab (Mabthera). Building trust in biosimilars is a vital component in this paradigm shift. Real-world clinical data will be an important next step in instilling trust in healthcare providers. King Abdulaziz Medical City Jeddah (KAMC-J) is currently conducting many real-world evidence studies of extrapolated indications on the use of oncology biosimilars [17]. One of our published extrapolated indication studies [18] and the preliminary data of many other unpublished studies are reassuring regarding the comparability of the efficacy and safety of oncology biosimilars in extrapolated indications of oncology biosimilars.

Infusion-related reactions (IRRs) are expected after rituximab administration, and can be life threatening. Therefore, it is recommended that all patients first receive at least one full dose of a rituximab product by intravenous infusion without experiencing severe adverse reactions before starting treatment with SC rituximab. If patients are not able to receive one full dose by intravenous infusion, they should continue subsequent cycles with a rituximab product by intravenous infusion and not switch to SC rituximab until a full intravenous dose is successfully administered [10,11,19].

At the Ministry of National Guards Health Affairs (MNGHA), an initial IV rituximab biosimilar is used, and if no severe IRRs are reported, subsequent cycles are administered using SC rituximab, per institutional guidelines [17]. There is currently no safety data available regarding this switch; however, many centers in the UK and Canada have already adopted this practice based on the extrapolation and switchability principles of biosimilar indications, which are approved by international regulators [20]. The MNGHA also approved this practice based on our institutional guidelines [21].

This study aimed to evaluate the real-world safety and efficacy of a rituximab biosimilar (Truxima) compared to the originator, as well as the IV-to-SC combined strategy of rituximab biosimilars in patients with B-cell lymphoma, focusing on IRRs. While clinical trials support the IV-to-SC switch for originator rituximab, real-world data on biosimilars are lacking.

## 2. Materials and Methods

### 2.1. Study Design and Patient Population

A retrospective observational study was conducted at the Princess Nourah Oncology Center (PNOC), King Abdulaziz Medical City, in Jeddah, Saudi Arabia. Following receipt of approval from the Institutional Review Board (IRB No. IRB/1539/23), electronic medical records were reviewed for eligible patients treated between October 2022 and June 2023. The objective was to assess the safety of IV rituximab biosimilar during the first cycle, followed by the administration of SC rituximab in the second cycle. Eligible patients were adults (≥18 years) diagnosed with follicular lymphoma, low-grade lymphoma, or diffuse large B-cell lymphoma (DLBCL). They must have received IV rituximab biosimilar (Truxima) during the first treatment cycle, followed by SC rituximab (Mabthera) in subsequent cycles at the PNOC. Patients were excluded if they had incomplete medical records, received only IV rituximab (Truxima), or were under 18 years of age.

### 2.2. Study Outcomes and Data Collection

The primary endpoint was the safety of IV rituximab biosimilar (Truxima-Biosimilar), assessed by the proportion of patients who developed an IRR after the first cycle. The severity of IRRs was graded using the Common Terminology Criteria for Adverse Events Version 5 (CTCAE.V5), which categorizes reactions into five grades [22]. Grade 1 indicates a mild, transient reaction that does not necessitate infusion interruption. Grade 2 refers to a reaction that requires symptomatic treatment or temporary interruption of infusion, with rapid symptom resolution. Grade 3 includes a prolonged reaction that does not respond quickly to treatment and may require hospitalization. Grade 4 involves life-threatening symptoms requiring immediate medical intervention, while Grade 5 corresponds to death resulting from the reaction. To our knowledge, this was the first real-world evidence study addressing the safety of the practice of administering a first cycle of IV rituximab biosimilar followed by SC rituximab in this extrapolated indication of rituximab biosimilar, and hence our key focus was the safety endpoints. Data were collected retrospectively from the patients’ electronic medical records and entered into a pre-designed Excel sheet in a de-identified manner. The secondary endpoint was the effectiveness of rituximab biosimilar, as assessed through the overall response rate (ORR) based on positron emission tomography/computed tomography (PET/CT) findings. Complete response (CR) was defined as a Deauville score of 1, 2, or 3; partial response (PR) as a score of 4; and progressive disease (PD) as a score of 5. The ORR was calculated as the combined proportion of patients achieving CR and PR.

### 2.3. Statistical Analysis

Continuous variables were reported as the mean with standard deviation (SD), while categorical data were presented as frequencies and percentages. Data were entered into Microsoft Office Excel and analyzed using GraphPad Prism software (version 10.0).

### 2.4. Use of Generative Artificial Intelligence (GenAI)

During the preparation of this manuscript, the authors used ChatGPT 4.0 Mini to paraphrase the text and enhance readability. In addition, the authors reviewed and edited the content as needed and take full responsibility for the content of this publication.

## 3. Results

### 3.1. Baseline Characteristics

A total of 71 patients were screened who had received IV biosimilar rituximab. Thirty-six patients were excluded from this study because they received IV rituximab biosimilar as a part of the salvage regimen for relapsed/refractory lymphoma (n = 20) and were not transitioned to SC rituximab, or they received IV rituximab biosimilar in CD20 + ve B cell acute lymphomablastic leukemia (n = 16) as a part of induction and consolidation, for which SC rituximab is not used. Only 35 patients met the eligibility criteria and were included in the final analysis. The majority were male (63%), with a mean age of 55 ± 18 years and an average weight of 73 ± 18 kg. Laboratory parameters relevant to the risk of infusion-related reactions included a mean white blood cell (WBC) count of 6 ± 3 × 10^9^/L and an absolute neutrophil count (ANC) of 4 ± 3 × 10^9^/L. Electrolyte levels, including potassium and calcium, were within normal ranges, with mean values of 4 ± 1 mmol/L and 2 ± 0.1 mmol/L, respectively. Common comorbidities included hypertension (43%), diabetes mellitus (31%), and cardiovascular disease (20%), while 45% of patients were medically free. The most frequent lymphoma diagnosis was diffuse large B-cell lymphoma (63%), followed by follicular lymphoma (20%). The most administered treatment regimen was R-CHOP (63%) followed by R-B (20%). The baseline characteristics of these patients are presented in Table 1.

### 3.2. The Incidence of IRRs

**Figure 1:**
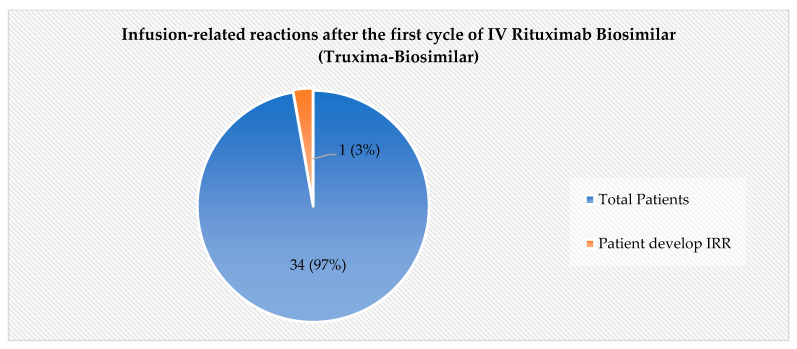
Infusion-related reactions (IRRs) after the first cycle of IV rituximab biosimilar (Truxima-Biosimilar).

In the assessment of the primary safety endpoint, the incidence of IRRs was found to be low. Among the 35 patients included in the final analysis, only one patient (3%) experienced a Grade 1 IRR, as per CTCAE.V5, during the first cycle of intravenous rituximab biosimilar (Truxima), as shown in Figure 1. The patient who developed a Grade 1 IRR eventually completed the intravenous infusion of rituximab (biosimilar) and successfully transitioned to subcutaneous rituximab for the subsequent cycle.

### 3.3. Effectiveness Outcomes

The effectiveness of the rituximab biosimilar was evaluated in 33 patients with available PET/CT results at the end of therapy. Among these, 79% (n = 26) achieved a CR, while 6% (n = 2) had a PR. In contrast, PD was observed in 15% of patients (n = 5). The ORR was 85% (n = 28), indicating a favorable treatment response in this cohort. Treatment responses are summarized in Table 2.

## 4. Discussion

Rituximab remains a cornerstone treatment for non-Hodgkin’s lymphoma, chronic lymphocytic leukemia, and even rheumatoid arthritis. It was the first monoclonal antibody approved by the FDA as an anticancer agent. The introduction of biosimilars, such as Truxima, offers a cost-effective alternative to the originator product, potentially increasing access to this critical therapy [6,23].

It is recommended that the patient be given one full IV dose before transitioning to the SC formulation. At the MNGHA, an initial IV rituximab biosimilar is used, and if no severe IRRs are reported, subsequent cycles are given using SC rituximab, per institutional guidelines. There are currently no safety data available for this switch; however, the KAMC-J was the first institution to retrospectively evaluate the safety of this practice and to design and complete a real-world evidence study of this practice for B-cell lymphoma. Results of that study demonstrated that only 1 of 34 patients developed an IRR; however, it was Grade 1, as per Common Terminology Criteria for Adverse Events v5.0, and the patient was able to complete the IV rituximab infusion in the first cycle. That study provided the first evidence that the transition from IV rituximab biosimilar to SC rituximab (Mabthera) is well tolerated and a safe practice, and recommended its implementation at other institutions.

The findings of our study indicate that the transition from IV to SC rituximab is well tolerated and safe. Only 1 (2.8%) of 35 patients developed an IRR, which was classified as Grade 1 according to the CTCAE.V5. This patient was able to complete the IV infusion successfully and subsequently transitioned to SC administration without further complications. This low incidence of IRRs is consistent with the safety profile observed in clinical trials of rituximab biosimilars [8,9].

Several studies have demonstrated the equivalent efficacy and safety of rituximab biosimilars compared to the originator product. For instance, the REFLECT trial reported a 94.7% ORR with Riximyo (a rituximab biosimilar) in combination with R-CHOP for CD20-positive diffuse large B-cell lymphoma (DLBCL) [8]. Similarly, the ASSIST-FL study found that Rixathon (another rituximab biosimilar) was equally effective and as safe as the originator rituximab in patients with advanced follicular lymphoma [9].

In our study, the secondary endpoint analysis showed that 79% of patients achieved a CR, defined as a Deauville score of 1, 2, or 3. This high response rate further supports the efficacy of rituximab biosimilars in real-world settings. The transition from IV to SC administration offers several advantages, including reduced infusion times and improved patient convenience, without compromising safety or efficacy.

The safety profiles of IV and SC rituximab are comparable, although IV administration is associated with a higher incidence of IRRs, particularly during the first infusion. On the other hand, SC administration may cause local injection-site reactions, such as pain or erythema [24,25]. The SC formulation is more convenient, taking only a few minutes to administer compared to over an hour for IV, thus reducing bed time and healthcare resource use [9].

Although biosimilars offer significant cost saving in general, cost saving impact was not expected to be very significant in our study, as rituximab biosimilar was used only in the first cycle before transitioning to SC rituximab. Therefore, we did not evaluate the cost-saving impact of using biosimilar rituximab.

Most centers in Europe and North America have already adopted the approach of a first cycle administration of IV rituximab biosimilar followed by SC rituximab. One of the strengths of our study was that it provided real-world evidence on the safety and efficacy of this practice, which is essential for informing clinical practice. Our study provides assurance of the safety of the practice of first cycle IV rituximab biosimilar followed by SC rituximab SC, which is an extrapolated indication. However, our study had some limitations. The sample size was relatively small, and the study was conducted at a single center. Therefore, larger, prospective, multicenter studies are needed to confirm our findings and explore the potential cost savings associated with the use of rituximab biosimilars.

## 5. Conclusions

In conclusion, our study demonstrated that the transition from IV rituximab biosimilar to SC rituximab Mabthera is a well-tolerated and safe practice. This approach can potentially improve patient convenience and reduce healthcare resource utilization. We recommend that this practice be implemented on a larger scale and in other institutions to further validate our findings and to explore the potential benefits of rituximab biosimilars in clinical practice.

## Figures and Tables

**Table 1:** Patients’ characteristics.

Characteristics	Overall (N = 35)
**Gender**	
Male	22 (63%)
Female	13 (37%)
**Weight (kilograms)**	73 ± 18
**Age (years)**	55 ± 18
**Laboratory values**	
Serum creatinine (µmol/L)	70 ± 31
Alanine aminotransferase (U/L)	18 ± 13
Aspartate aminotransferase (U/L)	28 ± 36
Platelet (×10^9^ g/L)	273 ± 126
Hemoglobin (g/dL)	12 ± 2
Hematocrit (%)	36 ± 6
White blood cells (×10^9^/L)	6 ± 3
Absolute neutrophils count (×10^9^/L)	4 ± 3
Potassium (mmol/L)	4 ± 1
Sodium (mmol/L)	138 ± 2
Calcium (mmol/L)	2 ± 0.1
Phosphate (mmol/L)	1 ± 0.2
**Comorbidities**	
Medically free	16 (45%)
Hypertension	15 (43%)
Diabetes mellitus	11 (31%)
Cardiovascular disease	7 (20%)
Hypothyroidism	4 (11%)
Benign prostatic hyperplasia	2 (6%)
Chronic obstructive pulmonary disease	2 (6%)
**Diagnosis**	
Diffuse large B-cell lymphoma	22 (63%)
Follicular lymphoma	7 (20%)
Lymphocyte-predominant Hodgkin lymphoma	3 (8%)
Primary mediastinal large B-cell lymphoma	2 (6%)
Splenic marginal zone lymphoma	1 (3%)
**Protocol**	
R-CHOP	22 (63%)
R-B	7 (20%)
R-GDP	3 (8%)
DA-R-EPOCH	2 (6%)
BR-Pola	1 (3%)

Numbers are presented as mean ± standard deviation or frequency with (percentage). R-CHOP: cyclophosphamide, doxorubicin, vincristine, prednisone, and rituximab; DA-R-EPOCH: dose-adjusted etoposide, prednisone, vincristine, cyclophosphamide, doxorubicin, and rituximab; R-GDP: gemcitabine, dexamethasone, cisplatin or carboplatin, and rituximab; BR-Pola: polatuzumab vedotin, bendamustine, and rituximab; B-R: bendamustine and rituximab.

**Table 2:** Response rates.

Response	Overall (n = 33)
Complete response	26 (79%)
Progressive disease (PD)	5 (15%)
Partial response	2 (6%)
Overall response rate	28 (85%)

The values represent number (proportion).

## Data Availability

Data are available upon reasonable request from the corresponding author.
